# Successful treatment of *low-flow-low-gradient* aortic stenosis and complex coronary artery disease in a patient with severely depressed left-ventricular function by protected percutaneous coronary intervention and transfemoral transcatheter aortic valve replacement via Y-graft vascular prosthesis: a case report

**DOI:** 10.1093/ehjcr/ytag035

**Published:** 2026-01-24

**Authors:** Yusuf Nejahsie, Samer Hakmi, Stephan Willems, Wolfgang Tigges, Eike Tigges

**Affiliations:** Department of Cardiology and Critical Care, Asklepios Klinik St.Georg, Lohmühlenstr. 5, Hamburg 20099, Germany; Department of Cardiac Surgery, Asklepios Klinik St.Georg, Lohmühlenstr. 5, Hamburg 20099, Germany; Department of Cardiology and Critical Care, Asklepios Klinik St.Georg, Lohmühlenstr. 5, Hamburg 20099, Germany; DZHK (German Center for Cardiovascular Research), partner site Hamburg/Kiel/Lübeck, Berlin, Germany; Department of Vascular Surgery, Asklepios Westklinikum Hamburg, Suurheid 20, Hamburg 22559, Germany; Department of Cardiology and Critical Care, Asklepios Klinik St.Georg, Lohmühlenstr. 5, Hamburg 20099, Germany; DZHK (German Center for Cardiovascular Research), partner site Hamburg/Kiel/Lübeck, Berlin, Germany

**Keywords:** Ischaemic cardiomyopathy, Valvular cardiomyopathy, Low-flow-low-gradient aortic stenosis, Y-graft vascular prosthesis, Protected PCI, TAVR, Case report

## Abstract

**Introduction:**

Low-flow, low-gradient aortic stenosis (LFLG AS) is a subset of aortic stenosis associated with a poor prognosis and high operative risk, particularly in the presence of concomitant coronary artery disease (CAD) requiring intervention. In patients considered inoperable, minimally invasive approaches often remain the only alternative. However, there are limited data on treatment strategies and outcomes in patients with LFLG AS and severe ischaemic cardiomyopathy undergoing percutaneous coronary intervention (PCI) and transcatheter aortic valve replacement (TAVR), especially in cases with challenging transfemoral access.

**Case summary:**

An 87-year-old male presented with progressive dyspnoea and angina. Diagnostics revealed a non-ST-segment elevation myocardial infarction, acute heart failure, acute-on-chronic renal failure, LFLG AS (aortic valve area of 0.7 cm²), and a left ventricular ejection fraction of 10%. Coronary angiography showed severe CAD requiring revascularization. Due to excessive surgical risk, we planned a staged interventional treatment by Heart Team consensus. First, high-risk PCI with mechanical circulatory support was performed, followed by transfemoral TAVR using a self-expandable 29-mm bioprosthesis via a femoral Y-graft conduit. The patient reported immediate relief of symptoms. Follow-up echocardiography at discharge and at 3 months showed improvement of the aortic valve function and an increase of the ejection fraction to 35%. The patient remained asymptomatic and resumed his daily activities.

**Discussion:**

This case demonstrates that Impella®-supported PCI prior to transfemoral TAVR is feasible and safe, even in the presence of a femoral Y-graft, in a patient with LFLG AS and severely reduced ejection fraction.

Learning points

- This case highlights the feasibility of transfemoral TAVR through a Y-graft vascular prosthesis, but requires a preprocedural assessment of the access route using multiple imaging modalities.
- The Impella CP can provide haemodynamic support during high-risk PCI and is feasible before addressing aortic stenosis.

## Introduction

*Low-flow, low-gradient* aortic stenosis (LFLG AS) is a subset of aortic stenosis (AS) associated with a poor prognosis and high operative risk.^1^ This condition occurs when left ventricular (LV) systolic dysfunction coexists with severe AS, resulting in a reduced stroke volume index (SVI). Transcatheter aortic valve replacement (TAVR) is a well-established alternative to surgical aortic valve replacement, especially in patients considered inoperable or at high surgical risk. However, TAVR alone may not be sufficient to improve outcomes in patients with LFLG AS and concomitant coronary artery disease (CAD), as residual myocardial ischaemia may limit the recovery of LV function and increase the mortality risk.^1^

Percutaneous coronary intervention (PCI) in patients with AS and severely reduced LV ejection fraction (LVEF) is challenging. In this context, the use of mechanical circulatory support devices such as the Impella® has been shown to be safe and effective in cases of cardiogenic shock, high-risk PCI, and acute myocardial infarction.^2^ However, evidence supporting its use in patients with LFLG AS and severe ischaemic cardiomyopathy undergoing PCI and TAVR remains limited.^3^

Transfemoral access is the most commonly utilized approach for TAVR with a favorable safety profile.^4^ However, data on large-bore access in Y-graft bioprostheses remain limited.^5^

Here, we present a case of successful treatment of a patient with LFLG AS and severe ischaemic cardiomyopathy by staged Impella®-supported PCI and transfemoral TAVR via an aortofemoral bypass graft.

## Summary figure

Educational Overview

**Table:** 

CLINICAL CASE OVERVIEW:	Patient Profile: 87-year-old male with LFLG AS and severe ischaemic cardiomyopathy. Initial Presentation: Progressive dyspnoea and angina, diagnosed with NSTEMI, acute heart failure, and acute-on-chronic renal failure. Diagnostic Findings: Valve area 0.7 cm², mean aortic valve gradient of 20 mmHg, LVEF 10%. Severe stenoses in left main, left anterior descending, and right coronary arteries. Surgical Risk: High operative risk (EuroSCORE 14.9%, STS 9.7%) and porcelain aorta; deemed unsuitable for surgery.
Treatment Strategy:	Day 1: Protected PCI High-risk PCI using Impella CP® via aortoiliac bypass graft. Successful revascularization with drug-eluting stents. Day 2: Transfemoral TAVR TAVR with 29-mm EVOLUT™ Pro Plus bioprosthesis. Transfemoral access via Y-graft; unilateral secondary access (≥2 cm distal to the delivery sheath); valve deployment under fast pacing.
Post-procedure Outcome:	Immediate symptom relief; improved LV function (20%). Discharged on Day 6 with optimal medical therapy. Follow-up after 3 month with further improvement of LV function (35%); NYHA II and CCS 0
Discussion Points:	Feasibility of staged Impella-supported PCI and TAVR in LFLG AS. Challenges and considerations in PCI for patients with severe AS and LV dysfunction. Safety access in TAVR via femoral Y-graft Valve-type selection.
Conclusion:	This case illustrates the feasibility and safety of a staged Impella-supported PCI before addressing the AS via transfemoral TAVR in spite of the presence of a femoral Y-graft in a patient with *LFLG AS* and severe ischaemic cardiomyopathy.

## Case presentation

An 87-year-old man with a history of hypertension, chronic kidney disease, atrial fibrillation, and peripheral arterial disease with bilateral aortoiliac bypass grafting (since 2012) was admitted due to progressive dyspnoea and angina. Upon admission, elevated serum levels of high-sensitive troponin (hsTnI) were identified, with a value of 548 ng/L (<34 ng/L),^6^ decreasing to 299 ng/L within a 3-h span. NT-proBNP was elevated to 12 803 ng/L (<450 ng/L),^6^ and creatinine to 2.6 mg/dL (0.7–1.2 mg/dL),^7^ with a baseline of 1.4 mg/dL due to chronic kidney disease. The ECG showed atrial fibrillation with a normal ventricular rate and absence of ST-segment elevations. Chest X-ray indicated pulmonary venous congestion. These findings were consistent with a non-ST-segment elevation myocardial infarction (NSTEMI) with heart failure and acute-on-chronic renal failure.

**Figure 1:**
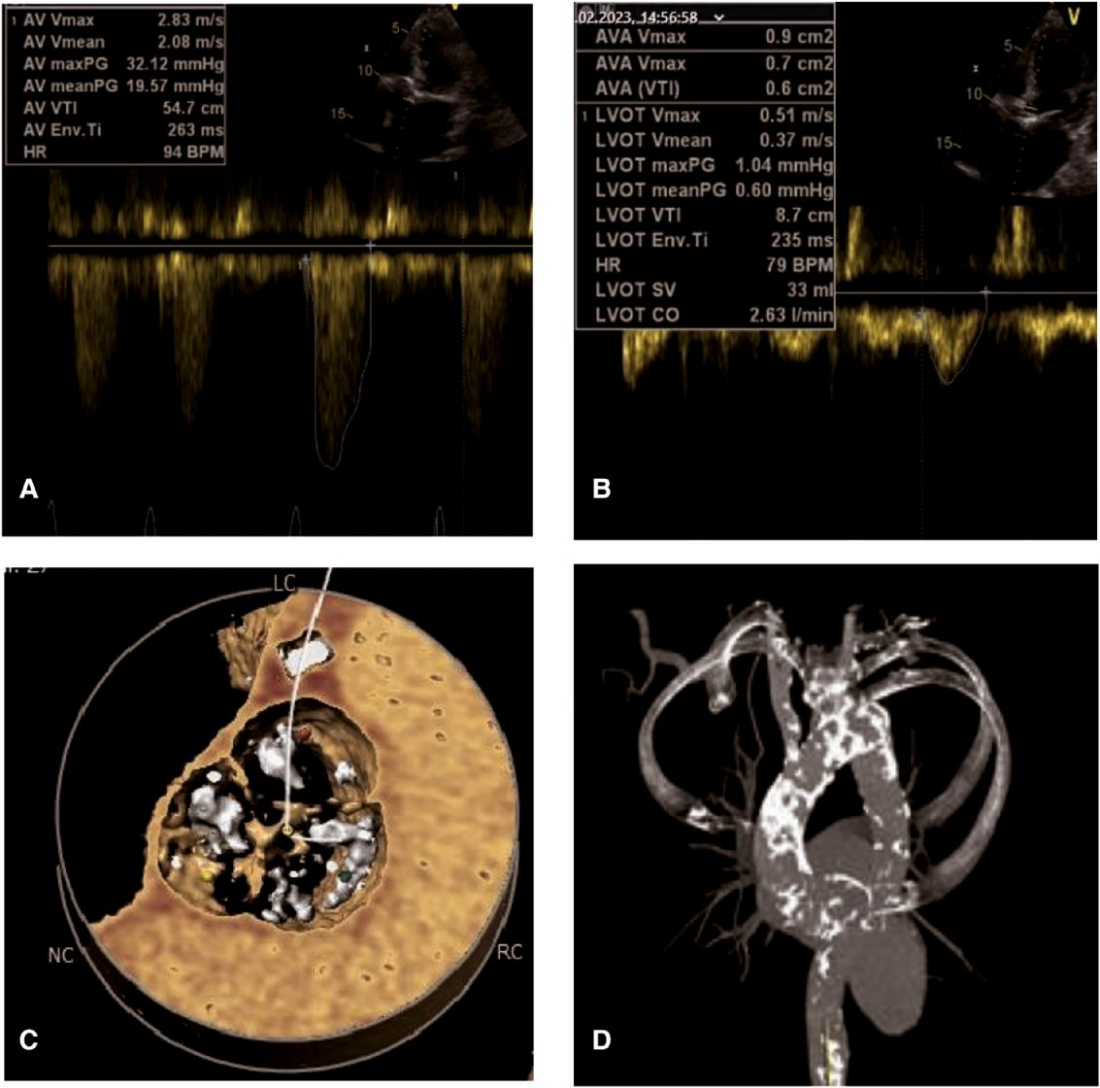
*(A+B)* Transthoracic echocardiography shows gradient measurements with a peak velocity of 2.83 m/s, a mean gradient of 19.57 mmHg, aortic valve area (measured by velocity time integral [VTI]) 0.6 cm^2^, and stroke volume (SV) 33 mL. *(C)* Hockey puck (VR)—Preprocedural CT scan showing severe aortic valve calcification. *(D)* Electrocardiography-gated cardiac computed tomography demonstrates severe calcification of the aorta ascendens–porcelain aorta.

**Figure 2:**
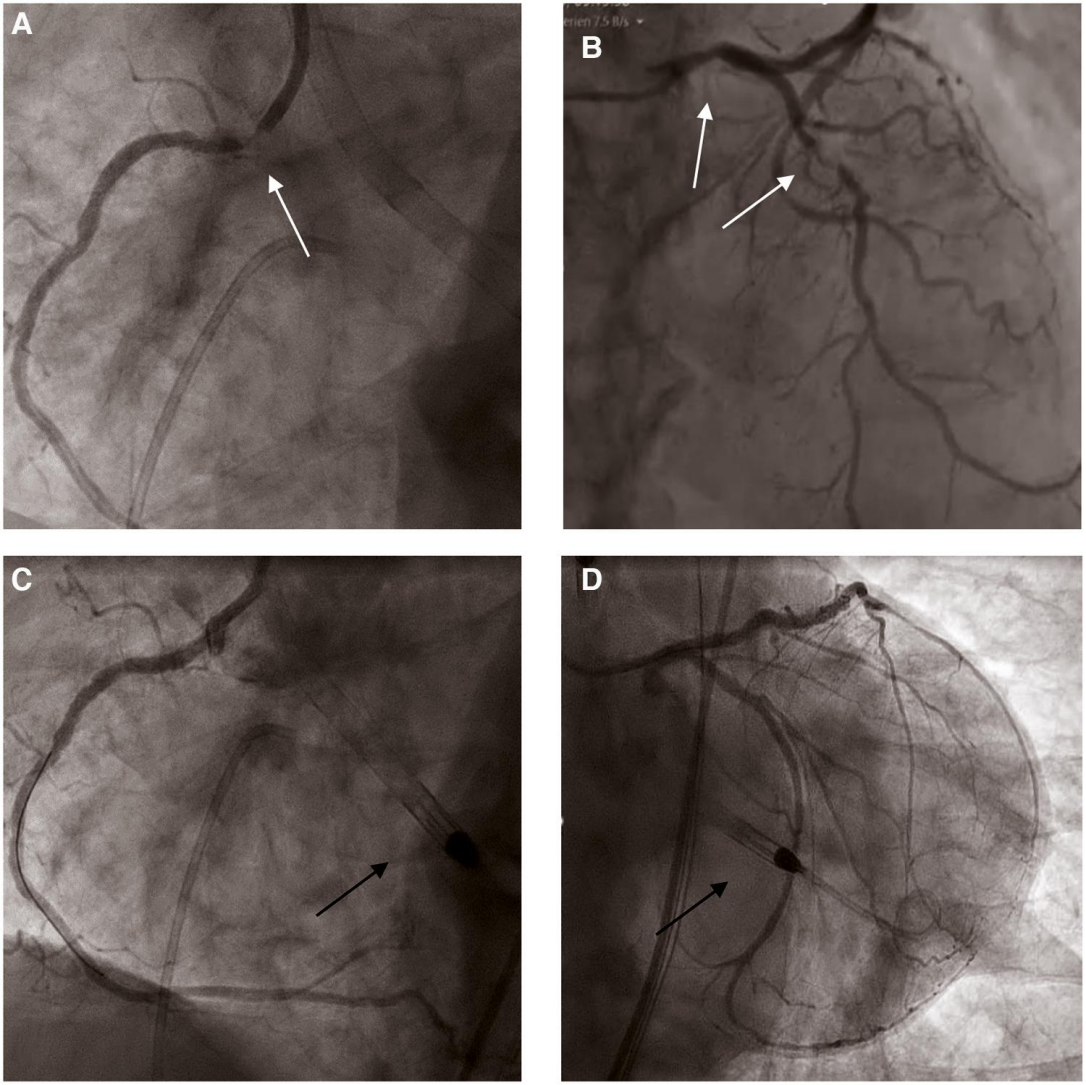
*(A and B)* Coronary angiography shows severe stenosis of the left main, left anterior descending, and the right coronary artery. White arrows show the stenosis. *(C and D)* Final angiographic results after implantation of drug-eluting stents in the left main, left anterior descending artery, and the right coronary artery. Black arrows show the correctly positioned Impella CPR device (Abiomed, Danvers, MA, USA) in the left ventricular.

Further diagnostics demonstrated a mean aortic valve gradient of 20 mmHg, and a LVEF of 10%, resulting in an aortic valve area (AVA) of 0.7 cm² (*Figure 1*; Supplementary material online, *Video S1*–*S3*). Coronary angiography showed severe stenoses in the left main (LM), left anterior descending (LAD), and an ostial stenosis of the right coronary artery (RCA; *Figure 2A* and *B*; Supplementary material online, *Video S4* and *S5*).

Given the patient’s clinical status and operative mortality risks (EuroSCORE 14.9%, STS 9.7%), the Heart Team recommended staged PCI followed by transfemoral TAVR. The patient provided informed consent after detailed discussion of risks and alternatives. On the first day, protected PCI of the LM, LAD and the RCA was performed via a 6-Fr sheath through the right radial artery. The Impella CP® was inserted via the vascular graft under fluoroscopic guidance, advanced across the calcified aortic valve, and maintained at 3.6 L/min throughout the procedure. Complete revascularization with drug-eluting stents was achieved (*Figure 2C* and *D*; Supplementary material online, *Video S7* and *S8*). The patient remained stable without inotropic support, and the Impella CP® was removed immediately after the procedure.

**Figure 3:**
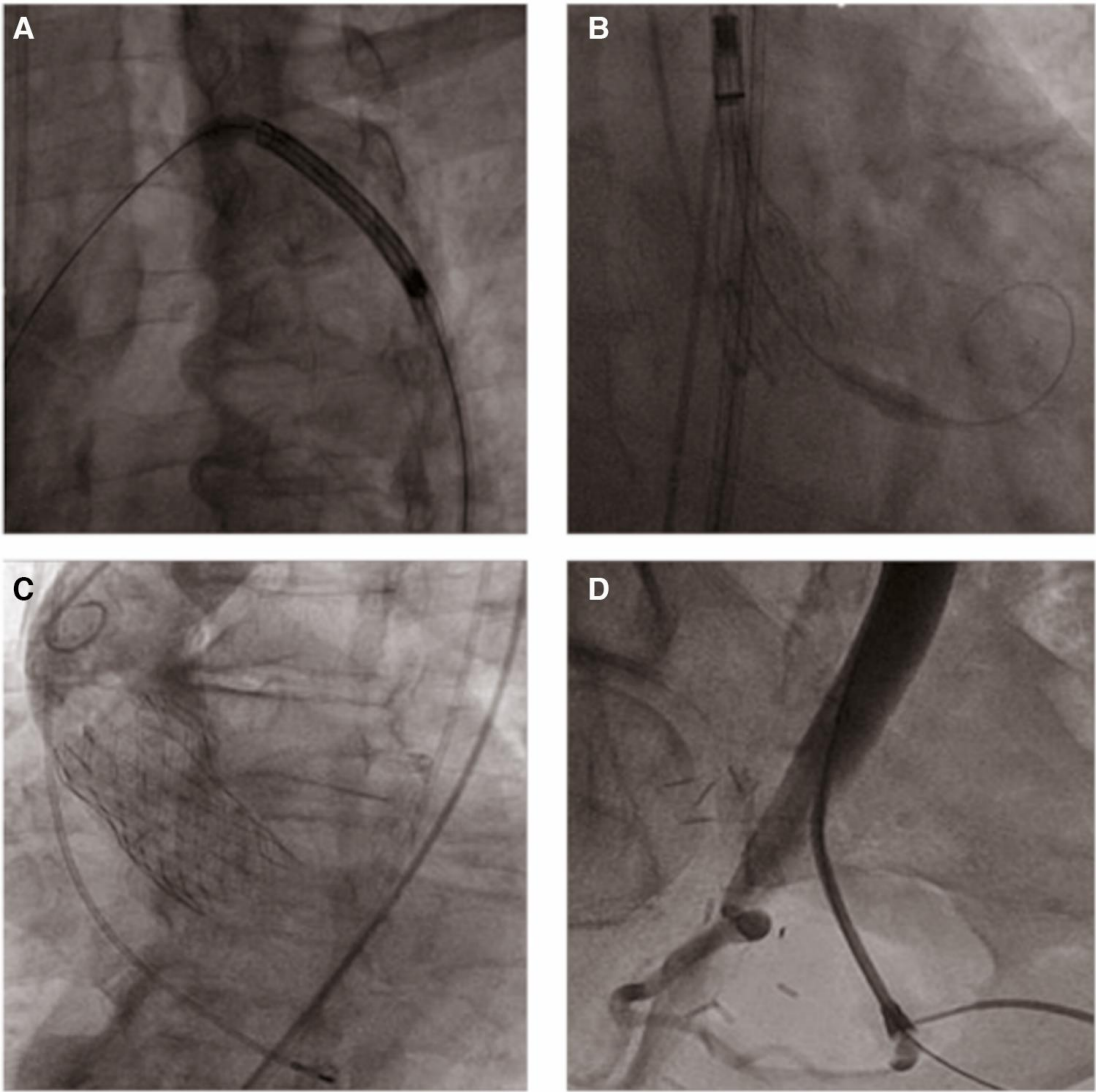
*(A)* Fluoroscopic image of crossing the aortic arch with the EVOLUT™ delivery system. *(B)* Deployment of the EVOLUT™ Pro+ in the cusp overlap view. *(C)* Final aortogram after successful implantation of EVOLUT™ Pro+ bioprosthesis without periprosthetic leaks. *(D)* Final peripheral angiogram confirming a patent Y-graft with preserved blood flow without any stenosis.

The following day, TAVR was performed via the right femoral Y-graft using a self-expandable 29-mm EVOLUT™ Pro+ bioprosthesis under local anaesthesia and sedation. Haemostasis was achieved with a suture-based vascular closure system, and final angiography confirmed a patent Y-graft and a well-functioning valve without significant leak or gradient (*Figure 3*; Supplementary material online, *Video S10* and *S11*).

Post-procedural monitoring was uneventful, without bleeding, renal impairment, or arrhythmias, including atrioventricular conduction disturbances. The patient was discharged on the sixth post-operative day with an improved LV function of 20% (see Supplementary material online, *Video S12*–*S14*).

An optimal heart failure medication regimen according to guidelines was initiated. At admission, the patient received a low-dose beta-blocker and a mineralocorticoid antagonist. Angiotensin receptor-neprilysin inhibitor and SGLT-2 inhibitor were added and titrated to maximum tolerated doses over 3 months. By then, LVEF improved from initially 10% to 35% (see Supplementary material online, *Video S15*–*S17*), symptoms improved to NYHA II/CCS 0, and NT-proBNP decreased from 12 803 to 5716 ng/L. Further follow-up could not be obtained, as the patient lives remotely and declined additional follow-ups.

## Discussion

The diagnosis of LFLG AS relies on the AVA, transvalvular gradient, SVI, and contractile reserve assessed by dobutamine-stress echocardiography.^8^ Due to ongoing unstable angina with heart failure, dobutamine stress echocardiography was not performed. However, the patient`s clinical presentation, severe aortic valve calcification (3360 Agatston units; *Figure 1C*), and planimetry of AVA supported the diagnosis of severe AS.

PCI in patients with LFLG AS and severe LV dysfunction poses challenges such as contrast-induced nephropathy, haemodynamic instability during balloon inflation, and stent deployment.^9^ An Impella CP® device was employed for mechanical circulatory support; although data remain limited in this population, smaller studies demonstrate its feasibility and safety.^10^ Regarding valve choice, balloon-expandable bioprostheses may facilitate coronary re-access. However, a self-expandable valve was chosen in this case to reduce the risks associated with rapid ventricular pacing due to the patient’s compromised haemodynamics and to better accommodate annular calcification with lower radial force, thereby minimizing rupture risk.

PCI was prioritized to address the urgency of NSTEMI management, avoid crossing the Impella CP® through a newly implanted valve, and mitigate risks of bioprosthetic valve damage or migration.

Complete revascularization was pursued, given the patient’s balanced coronary supply pattern in the setting of multivessel disease. The presenting symptom of angina was attributed to complex CAD, necessitating a comprehensive treatment strategy. Despite the patient’s advanced age and comorbidities, he had maintained an independent lifestyle until admission, when progressive angina and functional decline developed. Partial revascularization was unlikely to sufficiently reduce ischaemic burden, potentially impairing LV recovery and limiting TAVR benefits. Complete revascularization aimed to stabilize haemodynamics and reduce future coronary risks, especially due to challenging coronary access after TAVR with a supra-annular bioprosthesis. Overall, this strategy was chosen—taking into account the patient’s preference—to optimize both immediate and long-term clinical outcomes in this high-risk patient.

**Figure 4:**
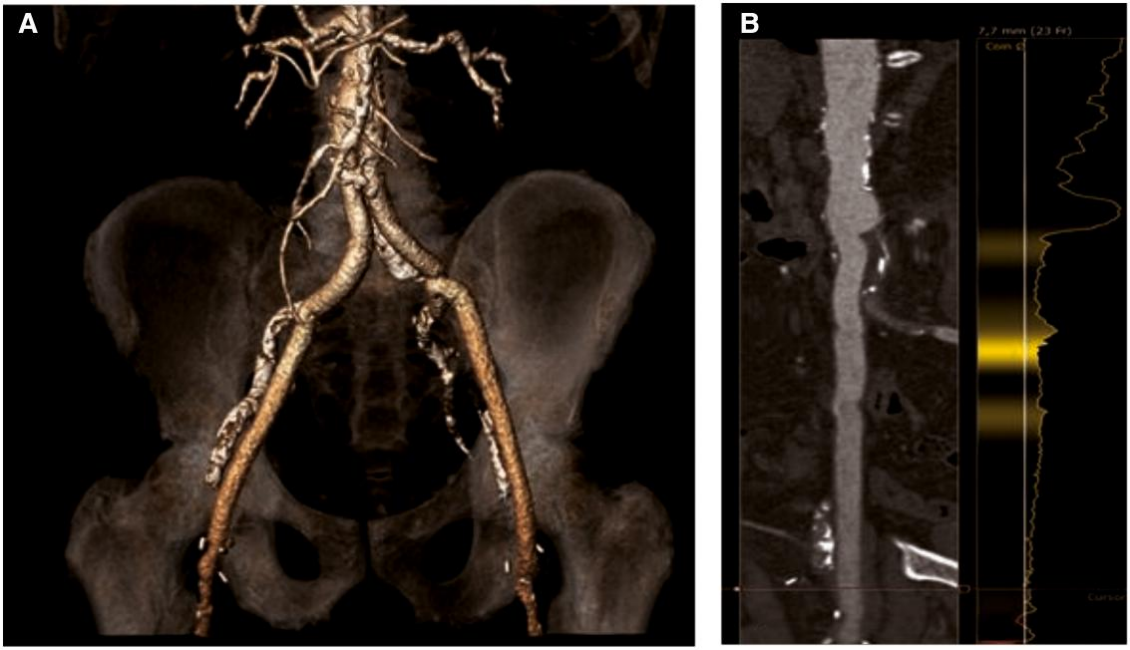
*(A)* Multislice CT (three-dimensional reconstruction) shows the bilateral aortoiliac graft and the femoral arteries. *(B)* Sufficient luminal diameter of the (right) Y-graft.

Limited evidence exists regarding transfemoral TAVR via prosthetic grafts. However, in our case, the decision to proceed transfemorally was supported by CT angiography, which confirmed a sufficient Y-graft diameter to safely accommodate a 14 Fr sheath (*Figure 4*). The presence of the Y-graft complicates contralateral safety access by rendering cross-over manoeuvres infeasible in case of vascular complications. Although alternative access routes such as apical, transaxillary, and transcaval approaches were discussed, these remain reserved for patients in whom transfemoral access is not feasible.^11^ To avoid cross-over manoeuvres, we used unilateral distal puncture (≥2 cm distal to the delivery sheath). Similar complex cases have been reported, with different management strategies. Soriano *et al*. presented a case series with TAVR followed by PCI without mechanical circulatory support,^12^ in contrast to our approach of Impella®-supported PCI prior to TAVR, which aimed to reduce ischaemic burden and avoid manipulating a freshly implanted valve with the Impella CP®. Naganuma *et al*. described transfemoral implantation of a balloon-expandable valve via surgical exposure of an aortofemoral graft.^13^ In contrast, we used a minimally invasive percutaneous graft puncture. To ensure vascular safety, ultrasound-guided puncture and pre-closure with ProGlide™ devices were performed. Our case also differed in valve type, use of circulatory support, and the complexity of complete revascularization. In summary, this case demonstrates the feasibility and safety of a staged approach with mechanical support, complete revascularization, and TAVR via complex access in high-risk patients.

## Lead author biography


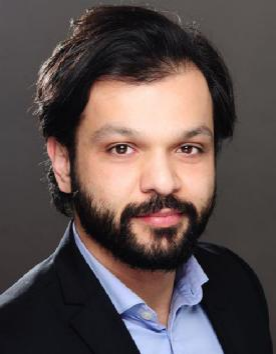


Dr Mohammad Yusuf Nejahsie graduated in 2018 from the Faculty of Medicine of the University of Hamburg, Germany. He is currently a Cardiology resident at the Department of Cardiology and Internal Intensive Care, Asklepios Klinik St. Georg in Hamburg-Germany. His main areas of interest are valvular and structural interventions.

## Supplementary Material

ytag035_Supplementary_Data

## Data Availability

All data underlying this article are available as part of the article. No additional source data are required.
